# Recent Trends in Pharmacological Activity of Alkaloids in Animal Colitis: Potential Use for Inflammatory Bowel Disease

**DOI:** 10.1155/2017/8528210

**Published:** 2017-01-09

**Authors:** Ana Cristina Alves de Almeida, Felipe Meira de-Faria, Ricardo José Dunder, Luis Paulo Bognoni Manzo, Alba Regina Monteiro Souza-Brito, Anderson Luiz-Ferreira

**Affiliations:** ^1^Institute of Biology, Department of Structural and Functional Biology, University of Campinas, Campinas, SP, Brazil; ^2^Faculty of Medical Sciences, Department of Pharmacology, University of Campinas, Campinas, SP, Brazil; ^3^Institute of Biotechnology, Department of Biological Sciences, Federal University of Goiás, Catalão, GO, Brazil

## Abstract

Inflammatory bowel disease (IBD) is a chronic and disrupted inflammation of the gastrointestinal tract. IBD have two main conditions, Crohn's disease and ulcerative colitis, and have been extensively investigated in recent years. Antibiotics derived from salicylates, steroids, immunosuppressors, and anti-TNF therapy are part of the therapeutic arsenal for IBD. However, very often patients stop responding to treatments over the time. In this context, searching for alternative agents is crucial for IBD clinical management. Natural products derived from medicinal plants are an interesting therapeutic alternative, since several studies have proven effective treatments in animal models of intestinal inflammation. Several naturally occurring compounds are potent antioxidants, both as free radical scavengers and as modulators of antioxidant enzymes expression and activity. A number of natural compounds have also been proved to inhibit the release of proinflammatory cytokines, decreasing the activation of nuclear factor *κ*B (NF-*κ*B), which is important to the inflammatory response in IBD. The alkaloids are substances of a very diverse class of plant secondary metabolites; an extensive list of biological activities has been attributed to alkaloids, such as being anticholinergic, antitumor, diuretic, antiviral, antihypertensive, antiulcer, analgesic, and anti-inflammatory. In the present work, studies on the pharmacological activity of alkaloids in experimental models of IBD were reviewed.

## 1. Introduction

Inflammatory bowel disease (IBD) is a chronic inflammatory disorder in the gastrointestinal tract and primarily includes two forms, ulcerative colitis (UC) and Crohn's disease (CD) [1]. UC is characterized by an inflammatory response with edema, ulceration and bleeding, and morphological changes along with the intestine mucosae, involving infiltration of polymorphonuclear cells (PMN), abscesses formation in mucosal crypts, and glands distortion. These changes are concentrated in the mucosa and restricted to the colon and rectum [2]. CD can affect any portion of the gastrointestinal tract, disturbing mainly the submucosa, but may be transmural and exceed the serosa, creating fistulas [3]. Etiology of IBD is complex and involves environmental factors, genetic factors, and an exacerbated immune response to commensal bacteria [4–6].

IBD was initially recognized as a health problem of developed countries; however, in a recent review, Molodecky and collaborators [7] have also reported increasing incidence and prevalence of IBD in developing countries, probably due to changes in lifestyles of these populations [8].

The therapy of IBD presents lack of effectiveness, high costs, and numerous side effects [9, 10]. Although anti-TNF*α* therapy revolutionized IBD clinical management, the number of reports showing loss of response in patients have been increasing. Moreover, almost one-third of IBD cases have not improved after anti-TNF*α* therapy [11]. Thus, the search for new alternative for the IBD therapy is still sine qua non.

For a century, natural products have been in the vanguard of drug discovery research, but, after the advent of automated high throughput screening (HTS) programs, the use of natural products in drug discovery in pharmaceutical industry declined [12]. However, natural products still continue to contribute to the drug development for cancer, infectious (bacterial, fungal, parasitic, and viral), immunological, cardiovascular, neurological, inflammatory, and related diseases [13, 14]. There is an increase in the number of IBD patients using complementary herbal therapies and many experimental studies and clinical trials present beneficial effect of vegetal extracts, fractions, or compounds [15].

In this work, we made a review in the studies on the effects of alkaloids, a class of plant compounds with several biological activities reported, in experimental intestinal inflammatory injury.

## 2. Alkaloids

**Figure 1:**
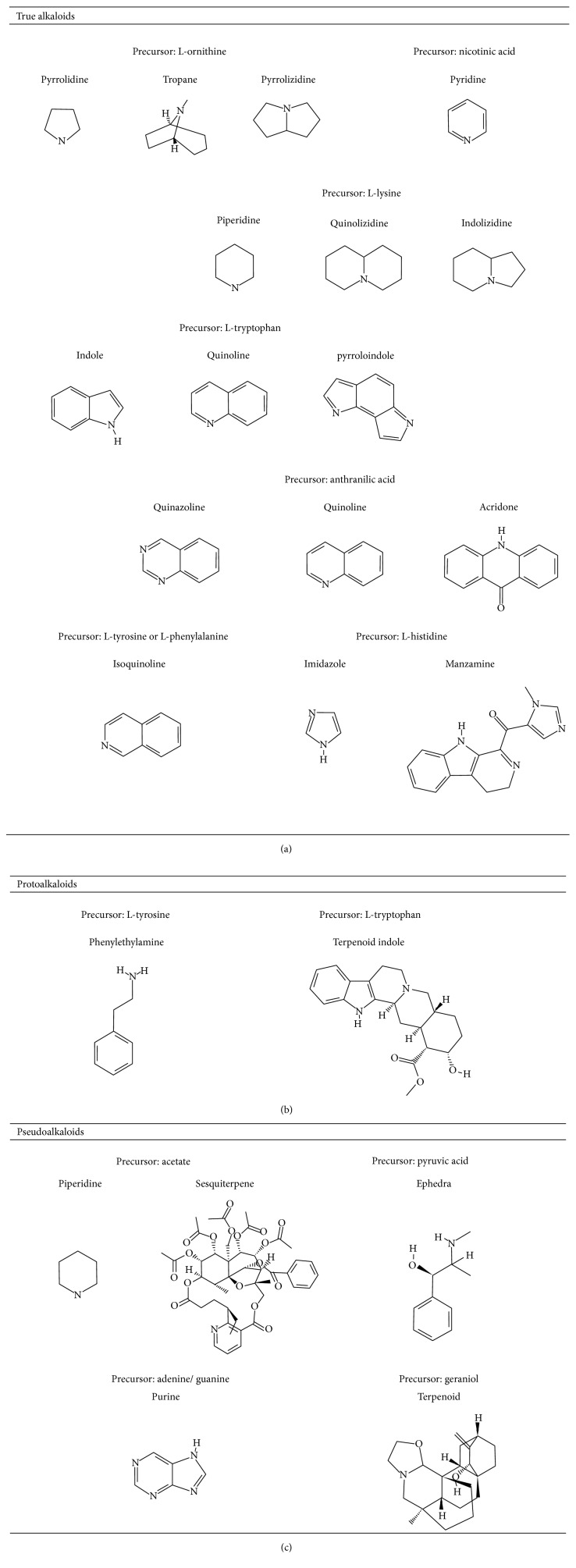
Main alkaloids groups: True Alkaloids (a), Protolakaloids (b) and Pseudoalkaloids (c), precursors, and skeleton structures. Adapted from Aniszewski, 2007 [18]. Chemical structures were getting in PubChem database.

The definition of the term alkaloid is not simple, but, in general, alkaloids are a group of natural nitrogen-containing basic compounds with low molecular weight, synthetized from amino acids and biologically active [16]. Alkaloids are a diverse group of compounds found in bacteria, fungi, plants, and animals [17]. There are three types of alkaloids: true alkaloids, protoalkaloids, and pseudoalkaloids. True alkaloids have a heterocyclic ring with nitrogen, while, in the protoalkaloids, the N atom derived from amino acids is not part of heterocyclic ring. Pseudoalkaloids are not originated from amino acids, including terpene-like, purine-like, and steroid-like alkaloids. The major groups of alkaloids are summarized in Figure 1.

The first alkaloids for medicinal use were isolated at the beginning of 19th century, by Derosne (opium salt, narcotine) and Sertürner (*principium somniferum, *morphine). The chemical identification of morphine was carried in 1923, by Robinson and Gulland [18, 19]. So far, there are more than 20000 alkaloids identified and a number of them have been placed an important role in clinical practice [20]. They present numerous biological activities such as being emetic, anticholinergic, antitumor, diuretic, sympathomimetic, antiviral, antihypertensive, analgesic, antidepressant, muscle relaxant, anti-inflammatory, antimicrobial, and antiulcer [21, 22]. The alkaloids have proton-accepting nitrogen atom and one or more proton-donating amine hydrogen atoms, which form hydrogen bonds with proteins, enzymes, and receptors. Furthermore, they, generally, have functional groups such as phenolic hydroxyl. The later might explain the exceptional bioactivity of the alkaloids [17].

Several studies have been demonstrating the anti-inflammatory activity of alkaloids, involving inhibition or regulation of important inflammation mediators such as NF-*κ*B, COX-2, and iNOS [23–29]. Souto et al. reviewed published studies to evaluate the anti-inflammatory activity of alkaloids and reported 40 of these compounds with significant activity [30]. Antioxidant activity of alkaloids have also been presented in different experimental models or pathological conditions [31–35]. Based on the chemical diversity of alkaloids and their biological activities previously reported, these compounds emerge as potential agents for intestinal inflammatory disorders.

## 3. Alkaloids and Experimental Colitis

This search was carried out on PubMed, Scopus, and Web of Science database, using the terms “Inflammatory Bowel Disease” OR “experimental colitis” OR “ulcerative colitis” OR “Crohn's Disease” OR “Colitis” AND “Alkaloid”. Publications over the last decade were considered.

### 3.1. Nicotine: Dual Role on IBD

Nicotine is studied as the main compound responsible for cigarette smoking properties on intestinal mucosa [36]. Smoking has an opposite effect in the two forms of IBD: while smoking increases the number and the risk of developing relapses in CD, in UC, surprisingly, the episodes of active disease are decreased [37, 38]. The effect of nicotine on intestinal inflammation has been widely studied in experimental assays [39–42] and clinical trials [43–45] and there are several reviews on the effects of nicotine in relation to IBD [46–49].

### 3.2. Plant Extracts and Herbal Formulation

The search shows some studies with plant extracts or herbal formulations, rich in alkaloids, used in traditional Chinese and Ayurvedic medicine as* Amaranthus roxburghianus*, Hangeshashinto, Fructus Mume pill, Sangrovit, and* Sophora alopecuroides*.

Our search on alkaloid and IBD has led to several plant extracts or herbal formulations commonly used in traditional Chinese and Ayurvedic medicine, such as Hangeshashinto (HST),* Sophora alopecuroides*, Fructus Mume pill (FMP), Sangrovit and, and* Amaranthus roxburghianus*.

Kawashima and colleagues [50] evaluated a combination of oriental medicinal plants, HST, in TNBS-induced colitis. Wistar rats treated with HST for 5 days presented decreased colon damage (macroscopic lesion score, ulcerative area, and colon weight), reduced diarrhea, and increased body weight. The authors also evaluated the effect of main constituents of HST, berberine (BE), baicalin (BA), glycyrrhizin (GL), and ginsenosides (GS) in the experimental TNBS colitis. These components were given alone, in combination (BA + BE and GL + GS), or in a total mixture (BA + BE + GL + GS). The compounds given alone and the combination BA + BE did not prevent colon injury, while GL + GS and the total mixture ameliorated the intestinal inflammation. The authors concluded that neither berberine nor baicalin is responsible for HST anticolitis effect; thus, HST was suggested to prevent or diminish the colitic phenotype due to the synergistic combination of its components.

Effects of total alkaloids of* Sophora alopecuroides* (TASA) were evaluated in TNBS-induced colitis in rats, by Chen and Deng [51]. TASA ameliorated histological damage, increased SOD activity, and decreased MDA levels, as well as NO and MPO activity. In a study from Zhou and colleagues [52], employing the same experimental model, the oral treatment with TASA decreased damage scores, maybe due to the upregulation of CD4+ CD25+ regulatory T cells (Tregs) and anti-inflammatory cytokine IL-10.

The effect of TASA on DSS-induced intestinal inflammation in mice has also been investigated. In this model, TASA inhibited acute inflammation in the gut by inhibiting the secretion of IL-1*β* and promoting the release of anti-inflammatory cytokine IL-4 [53]. Zhao et al. [54] assayed TASA treatment on DSS-induced chronic intestine injury. Chronically, TASA exhibited protective effects on DSS colitis inhibiting secretory immunoglobulin A and haptoglobin release; likewise, the intercellular adhesion molecule 1 (ICAM-1) gene expression and p65 recruitment to the ICAM-1 gene promoter were also found inhibited, suggesting that TASA might protect the intestine from injury by inhibiting NF-*κ*B activation.

FMP is a combination of ten Chinese herbs that has been used for a long time in traditional Chinese medicine for the treatment of diarrhea and dysentery; indeed, the State Food and Drug Administration of China approved its use for the management of gastrointestinal disorders. The effect of FMP was evaluated on TNBS-induced colitis in Sprague-Dawley rats. Remarkable results include reduced ulcer area, colon weight/length ratio, diarrhea, colonic MPO activity, INF-*γ* levels, gram −/gram + bacteria relation, and increased IL-4 levels [55]. The authors suggested that these effects may be related to the alkaloids aconitine and berberine. In another study, Zhang and colleagues [56] reported the synergistic activity of three alkaloids from FMP: berberine, hypaconitine, and skimmianine on TNBS-induced colitis in rats. Berberine ameliorated intestinal injury and diminished TNF-*α* levels and NF-*κ*B expression in colon but had no effect on abdominal pain (in acetic acid-induced writhing) nor gastrointestinal transit. Although hypaconitine and skimmianine did not exhibit anti-inflammatory activity on TNBS-induced colitis, the treatment with the combination of the three alkaloids ameliorated the colonic injury. The authors suggest that berberine exerted mainly anti-inflammatory activity, while hypaconitine possesses analgesic effect and skimmianine antidiarrheal properties.

Vrublova and colleagues [57] assayed the Sangrovit (rich in isoquinoline alkaloids) feeding supplementation in DSS-induced colitis in Wistar rats. Sangrovit is used as appetizer supplement in livestock feed. Sangrovit ameliorated histological damage score, decreasing COX-2 expression and colonic MPO activity. The alkaloids sanguinarine, dihydrosanguinarine, chelerythrine, and dihydrochelerythrine were found in the colon of Sangrovit-treated animals, suggesting a direct effect of these compounds in the colonic mucosae.

Iablokov and colleagues [58] evaluated the effects of potatoes glycoalkaloids on IL-10 knockout mice (genetic predisposition to develop colitis) and DSS treated mice. Their data show increased INF-*γ* levels in* ileum *of IL-10 knockout mice and increased intestinal permeability and INF-*γ*, IL-17, and TNF-*α* levels in DSS colitis mice. As a consequence, deleterious effects of glycoalkaloids were reported. The authors attributed such effects, mainly, to the compounds *α*-chaconine and *α*-solanine. The glycoalkaloids concentration can raise in about threefold in fried potatoes which has been shown to aggravate intestinal inflammation.

Nirmal and collaborators [59] analyzed the effect of* Amaranthus roxburghianus *root extract in combination with piperine on acid acetic-induced colonic injury in mice. The treatment with* A. roxburghianus* decreased histopathological damage, MPO activity, and MDA levels and major levels of reduced GSH compared to control (5% acetic acid). Interestingly, the combination with piperine improved the effects of the extract, similarly to those observed for prednisolone-treated animals while piperine itself had no effect.

In a recent publication, Bribi and colleagues [60] reported the protective effect of total alkaloids of* Fumaria capreolata* (AFC) on DNBS-induced colitis in mice. AFC treatment decreased the body weight loss, colon weight/length ratio, and microscopic score. AFC inhibited the increase of TNF-*α*, IL-1*β*, Il-6, IL-12, iNOS, ICAM-1, and MMP-9 and prevented the downregulation of MUC-2 mRNA expression in the colon. In LPS-stimulated CMT93 cells, AFC prevented upregulation of of ICAM-1, TNF-*α*, and IL-6 mRNA expression and the release of TNF-*α* and IL-6. In addition, ACF has also prevented the downregulation of of MUC-2 and ZO-1 expression in LPS-stimulated CMT93 cells. Stylopine, coptisine, and protropine were identified as major compounds in AFC.

### 3.3. Isolated Alkaloids

We found 32 alkaloids with activity assessed in experimental models that induced the disrupt of the epithelial barrier (DSS, acetic acid, or mustard oil) or that involved hapten-induced hypersensitive reactions in the intestinal inflammation (TNBS), mainly in mice.

In accordance with structural forms, the cited alkaloids are classified in diterpenoid alkaloids (14-O-acetylneoline, 14-O-veratroylpseudaconine, and hypaconitine), indole alkaloids (fumigaclavine C and isatin), indolonaphthyridine alkaloids (nigakinone), indoloquinazoline alkaloids (tryptanthin), isoquinoline alkaloids (berberine, boldine, cavidine, coptisine derivatives, EM012, papaverine, sanguinarine, sinomenine, tetrahydrocoptisine, and tetrandrine), phenanthroindolizidine alkaloids (NK-007 and W-8), piperidine alkaloids (piperine), purine alkaloids (caffeine), quinoline alkaloids (skimmianine), and quinolizidine alkaloids (matrine, oxymatrine, sophocarpine, and sophoridine). The effects of these alkaloids on experimental colitis are summarized in Table 1. Berberine, sinomenine, and piperine effects on experimental colitis were assessed in two or more manuscripts, as detailed hereafter.

Berberine has been the wieldiest studied alkaloid with pharmacological activity in intestinal inflammatory models. It is one of the main substances of* Berberis *sp. [95]. It is also isolated from* Hydrastis*,* Coptis,* and* Phellodendron *species [96]. The effect of berberine in colitis was evaluated in DSS and TNBS-induced colitis in mice and rats. This alkaloid ameliorates colon injury and inhibits the increase of inflammatory mediators and oxidative damage, as described in Table 1.

The treatment with berberine was used as reference drug for* Berberis vulgaris* fruit extract (BFE) treatment during the evaluation of its effects on acetic acid-induced colitis [65]. Both oral BFE and enema treatment were effective in diminishing the macroscopic and histopathological damage. As BFE is nearly disproved of berberine alkaloid, the authors attributed the effect to other components, such as anthocyanins. Kawashima and collaborators [50] reported no significant effect of berberine (3.75 or 6.5 mg·kg^−1^) treatment in TNBS-induced colitis. This result is probably due to the doses used, while the protective effect of berberine in experimental colitis was observed with doses of 10–100 mg·kg^−1^ [64, 66, 67, 69].

The addition of berberine (20 mg·kg^−1^) to 5-ASA treatment (200 mg·kg^−1^) decreased disease severity of colitis induced by DSS in mice. 5-ASA in combination with berberine decreased the disease activity index (DAI) and histological injury, inhibited COX-2, TNF-*α*, IL-12b, and IL-23 expression in colon tissue, and inhibited NF-*κ*B and JAK activation. Moreover, the combination of 5-ASA and berberine did not produce any deleterious effects in mice. The authors suggest that the combination is promising to UC therapy.

Berberine is an isoquinoline quaternary alkaloid and presents therapeutic properties, as antimicrobial, antidiabetic, anticancer, and anti-inflammatory, and has pharmacological activity in gastroenteritis, abdominal pain, and diarrhea [28, 31, 97]. Berberine also improves intestinal epithelial tight junction integrity, as presented by Gu et al. [98]. This study proposes reduced epithelial gut permeability as a possible mechanism of antidiarrheic activity of berberine. DiGuilio and colleagues [99] tested berberine treatment, in vitro, using CACO-2 cells barrier leaking induced by cytokines (TNF-*α*, IL-1*β*, and INF-*γ*) or hydrogen peroxide. Berberine enhanced basal CACO-2 barrier integrity and also decreased cytokine-induced injury in epithelial barrier function, suggesting that this phenomenon might also contribute to the role of berberine on experimental model of colitis.

The alkaloid oxymatrine activity in experimental colitis was reported in four articles [79–81, 83]. The treatment with oxymatrine improves TNBS and DSS-induced colitis. The protective effect of oxymatrine was also evaluated in intestinal injury induced by ischemia and reperfusion. The authors reported decreased apoptosis index, intestinal lipid peroxidation, serum TNF-*α* levels, phosphorylated p38 mitogen-activated protein kinase (MAPK), and Fas/FasL expression [100].

Sinomenine activity in experimental colitis was reported in two manuscripts [88, 89]. This alkaloid inhibits the generation of inflammatory mediators in TNBS-induced colitis in mice. In experimental model of colitis, sinomenine has been suggested to downregulate microRNA 155 (MiR-155), the transcription factor c-Maf, and the cytokines TNF-*α* and IFN-*γ*. Sinomenine has also demonstrated analgesic activity on neuropathic and inflammatory pain models [101], suppressive effect on colon carcinoma cell growth [102, 103], and anti-inflammatory activity [104].

Li and collaborators [105] employed the alkaloid piperine to ameliorate curcumin absorption and pharmacological activity. Piperine and curcumin were encapsulated in a nanoformulation, called self-microemulsifying drug delivery system (CUR-PIP-SMEDDS). The system CUR-PIP-SMEDDS increased the drug stability and the dissolution of curcumin at the colon site (in vitro) and showed therapeutic effects in DSS-induced colitis in mice. CUR-PIP-SMEDDS decreased DAI, histopathological lesions, MPO activity, MDA content, TNF-*α*, and IL-6 levels in colonic tissue of mice.

In a study from Hu et al. [85], the evaluation of the mechanisms of action of piperine on DSS-induced colitis allowed the development of siRNA-mediated knockdown of PXR in mouse colons and also indicated a role of PXR in protecting colonic mucosae. Piperine treatment prevented body weight loss, diarrhea, histological injury, and the expression of inflammatory mediators on DSS-induced colitis in mice. When the PXR was downregulated, the DSS injury was exacerbated and piperine protection against DSS colitis was inhibited.

We found two studies with comparative analysis of several alkaloids in experimental colitis. Wangchuk et al. [62] isolated five diterpenoids alkaloids (pseudaconitine, 14-veratroylpseudaconine, 14-O-acetylneoline, neoline, and senbusine A) of* Aconitum laciniatum,* a species of aconites of polyherbal formulations in Bhutanese Traditional Medicine, for inflammatory conditions. In this work, the authors reported the evaluation of 14-veratroylpseudaconine and 14-O-acetylneoline in TNBS-induced colitis in mice. The compound 14-veratroylpseudaconine exacerbated the TNBS-induced damage, while 14-O-acetylneoline ameliorated some signals of injury and inhibited the INF-*γ* release. In the manuscript of Zhang et al. [61], thirty synthesized coptisine derivatives were found to activate the in vitro transcription of x-box-binding protein 1 (XBP1). The dihydrocoptisines were demonstrated to be much more active antiulcerative colitis agents than quaternary coptisines and tetrahydrocoptisines, by in vitro XBP1 transcriptional activity assays and animal experiments (DSS-induced colitis). The authors also demonstrated that reductive states and the substitution patterns of the dihydrocoptisines are critical for their efficacy; unsubstituted dihydrocoptisine exhibited more significant efficacy in mice colitis than dihydrocoptisines substituted at the C-8 or C-13 position.

## 4. Discussion

In the present search, alkaloids of different groups showed protective activity in experimental colitis, involving distinct mechanisms. Some alkaloids did not present significant effects in colitis, whereas others, as potatoes glycoalkaloids, exhibited deleterious action in IBD. The majority of the alkaloids studied in experimental colitis were isolated from herbal formulations and plant preparations used in traditional eastern medicine. The evaluation of these extracts in experimental colitis showed their significant and beneficial activity. The latter isolation and study of the components of the effective extract identified some alkaloid with potential activity in IBD. Among them, the berberine was the most reported and the most cited in a review of alkaloid anti-inflammatory activity [30]. This manuscript related 40 alkaloids to active effects in different models of inflammation.

IBD therapy includes aminosalicylates, corticosteroids, immunosuppressive agents, and biological agents. Aminosacicylates and corticosterois do not provide long-term clinical response and mucosal healing and immunosuppressors do not induce remission [106]. The anti-TNF-*α* drugs infliximab and adalimumab improved IBD therapy. They have been shown to induce clinical and endoscopic remission in both CD and UC, to diminish exacerbations and surgery rates [107]. However, one-third of IBD patients are unresponsive to TNF-*α* antibodies and another third of patients become nonresponsive after a time of treatment. Efficacious treatment options for these patients are imperative [108].

Natural products can be a source of immune modulators antioxidants and anti-inflammatory substances [97, 109–111]. The berberine decreased colonic inflammation in UC and CD experimental models and inhibited cytokines release (TNF-*α*, IL-1*β*, IL-6, IL-12, and IL17). This effect can be promising to IBD therapy, but there are no clinical studies related to berberine in IBD. This isoquinoline alkaloid has been evaluated in different clinical trial studies and is showed to decrease symptoms of irritable bowel syndrome [112], nonalcoholic fatty liver disease [113], and acute coronary syndrome inflammation [114] without any side effects related to berberine treatment.

The combination of therapeutic drugs and alkaloids may be an approach of induction of remission, with fewer collateral effects. A minor dose of 5-ASA showed protective effect in mice DSS colitis, in a combination with berberine [115]. Abdel-Daim and collaborators highlighted the role of natural products in ameliorating collateral effects of standard drugs [116]. However, it is important to regard alkaloids toxicity and safety to human use.

The study of alkaloid properties in IBD may contribute to the development of new drugs. Considering the variety of structures and the biological effects of alkaloids, anti-inflammatory and antioxidant activity reported, and the small number of published articles, there is still much to be explored in this chemical class in IBD.

## Figures and Tables

**Table 1:** Summary of alkaloids effects in experimental colitis models.

Substance (class) and source	Experimental model	Results	Reference
*(±)-8-Acetonyldihydrocoptisine* (Isoquinoline) Derivative of coptisine, encountered in *Coptis *sp*., Corydalis *sp. 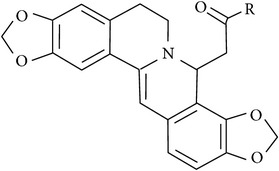	C57BL/6J mice DSS	↓ body weight loss ↓ DAI ↓ colon length shortening	[61]

*14-O-Acetylneoline* (Diterpenoid) *Aconitum laciniatum* 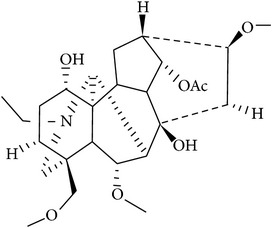	C57BL/6 mice TNBS	↓ body weight loss ↓ clinical score ↓ piloerection and mobility score ↓ faecal consistence score ↓ macroscopic damage score ↓ colon length decrease ↓ histological injury ↓ INF-*γ* secretion	[62]

*14-O-Veratroylpseudaconine* (Diterpenoid) *Aconitum laciniatum* 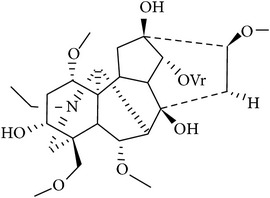	C57BL/6 mice TNBS	↑ inflammation (body weight loss)	[62]

*Berberine* (Isoquinoline) *Berberis vulgaris, B. aquifolium, Coptis chinensis, Coptis japonica, Hydrastis canadensis, Mahonia aquifolium,Phellodendron amurense* 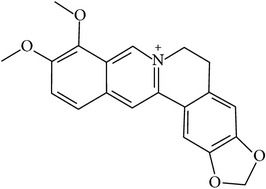	Sprague-Dawley rats TNBS	↑ body weight ↓ macroscopic damage score ↓ histological damage score ↓ MPO activity and IL-8 production	[63]
	C3H/NeH mice C3H/HeJ mice TNBS	↓ body weight loss and macroscopic damage score ↓ MPO activity, iNOS, MDA, and 4-HNE levels ↓ IL-1*β*, TNF-*α*, and IL-6 levels ↑ IL-10 and GSH levels, SOD, and CAT activity	[64]
	Sprague-Dawley rats TNBS	↓ colon index and ulcerative area ↓ TNF-*α* levels in colon tissue ↓ NF-*κ*B expression in colon tissue = TLR4 expression in colon tissue = LBP level in colon tissue ↓ LBP level in LPS treated cells (HT29 cells) = PGE_2_ level in LPS treated cells (HT29 cells) ↓ TNF-*α* level in LPS treated cells (HT29 cells)	[56]

*Berberine* (Isoquinoline) *Berberis vulgaris, B. aquifolium, Coptis chinensis, Coptis japonica, Hydrastis canadensis, Mahonia aquifolium, Phellodendron amurense* 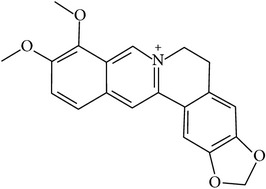	Wistar rats Acetic acid	↓ lesion score and ulcerative area ↓ colon weight/length ratio ↓ histopathological damage ↓ colitis index and ulcer index	[65]
	C57BL/6 mice DSS	↓ body weight loss ↓ inflammation score ↓ MPO activity ↓ TNF-*α*, INF-*γ*, IL-17, and KC levels ↓ TNF-*α* levels in colonic macrophages ↓ I*κ*B degradation, ERK1/2, and p38 activation in colonic macrophages and epithelial cells	[66]
	BALB/c mice DSS	↓ body weight loss ↓ spleen weight ↑ thymus weight ↓ blood hemoglobin levels reduction ↓ colonic MPO activity and MDA levels ↓ INF-*γ* and IL-12 in splenic lymphocyte production ↑ IL-10 and IL-4 in splenic lymphocyte production	[67]

*Berberine* (Isoquinoline) *Berberis vulgaris, B. aquifolium, Coptis chinensis, Coptis japonica, Hydrastis canadensis, Mahonia aquifolium, Phellodendron amurense* 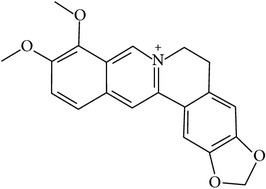	C57BL/6 mice DSS	↓ body weight loss ↓ colon length decrease ↓ lymphocytes infiltration ↓ INF-*γ* and IL-17 secretion (acute phase) Dopamine D1 and D2-like receptors antagonism ↓ INF-*γ*, TNF-*α*, and IL-6 secretion in splenic lymphocytes and peritoneal macrophages ↓ TNF-*α*, IL-6, IL-12, and TGF-*β* secretion in bone marrow dendritic cells (BMDC) ↑ IL-1*β* secretion in BMDC cells	[68]
	C57BL/6 mice DSS	↓ body weight loss ↓ stool consistence and bleeding scores ↓ DAI and histological injury score ↓ colon length decrease ↑ occludin, IL-4, IL-10, and Foxp3 expression ↓ IL-17, IL-6, IL-23, TNF-*α*, T-bet, ROR-*γ*t expression, and STAT3 activation ↓ Th17 cells infiltration	[69]

* Boldine* (Isoquinoline) *Peumus boldus* 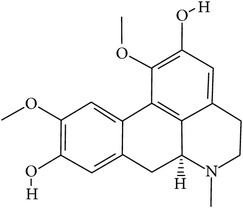	Balb/c mice DSS	↓ DAI and histological damage ↑ colon length and ↓ spleen weight ↓ MPO activity and MDA content in colon ↑ SOD and CAT activities in colon ↓ TNF-*α*, IL-6, IL-17, CD68^+^, and NF-*κ*B expression and pSTAT3 activation in colon ↑ I*κ*B-*α* expression in colon ↓ NF-*κ*B expression in nucleus of LPS-treated RAW 264.7 cells	[70]

** ** *Caffeine* (Purine) *Coffea arabica, Cola acuminata, Ilex paraguariensis, Paullinia cupana* 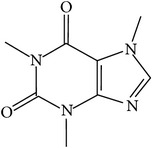	C57BL/6 mice DSS	↓ body weight loss ↓ clinical and histological scores ↓ tissue F4/80, CD4 and C11b positive cells and bacteria number ↓ TNF-*α*, INF-*γ* and IL-17b levels ↑ IL-10 and IL-4 levels ↓ AMCase and CHI3L1 expression ↓ Akt activation	[71]

* Cavidine* (Isoquinoline) *Corydalis impatiens* 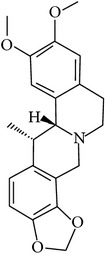	Kunming mice Acetic acid	↓ body weight loss and mortality ↓ DAI ↓ macroscopical and histological scores ↓ colon weight/length ratio ↓ NF-*κ*B, TNF-*α*, and IL-6 expression in colon ↓ TNF-*α* and IL-6 serum levels ↓ MPO activity and MDA levels in colon ↑ SOD activity and GSH levels in colon	[72]

* Dihydrocoptisine* (Isoquinoline) Derivate of coptisine, encountered in *Coptis *sp*., Corydalis *sp. 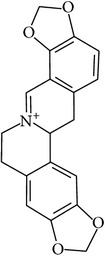	C57BL/6J mice DSS	↓ body weight loss ↓ colon length shortening ↓ DAI and macroscopic damage score ↓ histological damage	[61]^*∗∗*^

** ** *EM012 (reduced brominated derivative of noscapine)* (Isoquinoline) Derivative of noscapine, encountered in *Papaver somniferum* 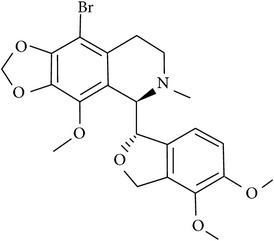	C57BL/6J mice DSS	↓ body weight loss ↓ colon length shortening ↓ histological damage ↓ MPO activity in colon ↓ IL-1*β*, IL-6, and INF-*γ* levels in colon	[73]

** ** *Fumigaclavine C* (Indole) *Aspergillus fumigatus* 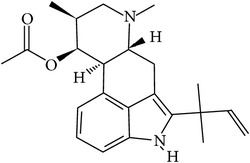	BALB/c mice TNBS	↓ macroscopic damage ↓ histological damage scores ↓ body weight loss ↓ mortality ↓ IL-1*β*, IL-2, IL-12*α*, IFN-*γ*, TNF-*α*, and MMP-9 mRNA levels ↓ IL-2 and IFN-*γ* level ↓ MMP-9 activity = COX-1	[74]

*Hypaconitine* (Diterpenoid) *Aconitum carmichaeli* 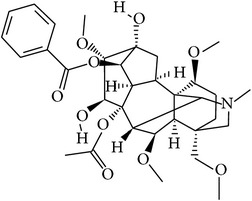	Sprague-Dawley rats TNBS	= colon index = ulcerative area ↓ LBP level in colon tissue = TNF-*α* levels in colon tissue ↓ NF-*κ*B and TLR4 expression in colon tissue ↓ LBP and PGE_2_ levels in LPS treated cells (HT29 cells) = TNF-*α* level in LPS treated cells (HT29 cells) ↑ frequency and amplitude of contract in colon and duodenum	[56]

* Isatin* (Indole) *Isatis *sp*., Calanthe discolor, Couroupita guianensis* 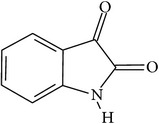	Wistar rats TNBS	↓ macroscopic score ↓ GPx and GR activity and ↑ SOD activity and GSH contents ↓ COX-2 expression = COX-1 expression ↓ TNF-*α* expression ↓ INF-*γ* and PGE_2_ levels ↑ IL-10 levels	[75]

** ** *Matrine* (Quinolizidine) *Sophora sp.* ** ** 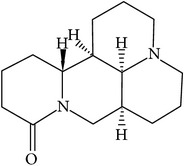	BALB/c mice TNBS	↓ body weight loss ↓ macroscopic and histological damage scores ↓ MPO activity ↓ TNF-*α* mRNA and protein levels	[76]

** ** *Nigakinone* (Indolonaphthyridine) *Picrasma quassioides* 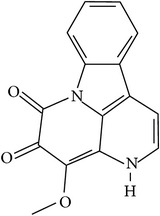	Wistar rats DSS	↓ body weight loss ↓ colon length shortening ↓ DAI ↓ MPO activity ↓ TNF-*α* serum levels	[77]

** ** *NK007 * (Phenanthroindolizidine) Derivative of tylophorine, encountered in *Tylophora *sp. 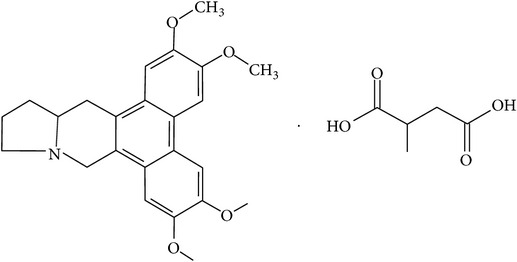	BALB/c (Tnf-*α*-luc)-Xen mice DSS (acute colitis)	↓ body weight and colon length loss ↓ TNF-*α* (luciferase activity) ↓ histological damage	[78]
	C57BL/6 mice DSS (chronic colitis)	↓ DAI and macroscopic damage scores ↓ histological damage, ↓ TNF-*α* and IL-12 levels ↓ *p*-p65 (NF-*κ*B) expression	
	Wistar rats Acetic acid	↓ DAI and macroscopic damage scores ↓ TNF-*α* and IL-12 levels and ↓ *p*-p65 (NF-*κ*B) and *p*-I*κ*B expression (in vitro)	

** ** *Oxymatrine* (Quinolizidine) *Sophora flavescens* 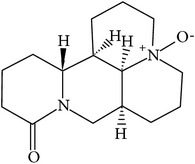	Sprague-Dawley rats DSS	↓ DAI and mucosal damage scores ↓ TNF-*α* and IL-6 serum levels ↓ colonic ICAM-1 and NF-*κ*B expression	[79]
	Sprague-Dawley rats TNBS	↓ body weight loss ↓ looser stool and bloody purulent stool ↓ macroscopic and histological score ↓ NF-*κ*B (p65) expression ↑ 2*β*AR and *β*-arrestin-2	[80]
	Sprague-Dawley rats TNBS	Attenuation of colitis (diarrhea, bloody stool, and histopathological changes were analyzed) ↓ *β*-arrestin-1 and Bcl-2 expression	[81]^*∗*^
	Rats TNBS	Inflammation amelioration ↓ IL-2 levels ↓ NF-*κ*B expression ↑ IL-10 levels	[82]^*∗*^
	C57BL/6 mice DSS	↑ body weight ↓ histological damage score ↓ IL-6 and IL-1*β* mRNA colonic levels	[83]

** ** *Papaverine* (Isoquinoline) *Papaver somniferum* 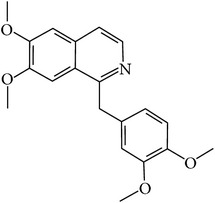	C57BL/6 mice DSS	= body weight loss ↓ DAI ↓ bleeding and diarrhea ↓ histological damage ↑ venular leukocyte adherence in colon = venular platelets adherence in colon	[84]

*Piperine* (Piperidine) *Piper nigrum, Piper longum* 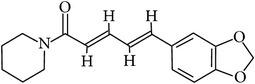	Swiss mice Aceti acid	= histological changes = MPO activity, MDA content, and GSH levels in serum and in colon	[59]
	C57BL/6 mice DSS	↓ body weight loss and diarrhea ↓ colon length decrease ↓ macroscopic and histological scores ↑ PXR, Cyp3a11, Cyp3a13, GST*α*1, and MDR1*α* mRNA expression in colon tissue ↓ ICAM, iNOS, IL-1*β*, MCP-1, IL-6, IL-10, and TNF*α* mRNA expression in colon tissue = CCR2 mRNA expression in colon tissue	[85]

*Piperine* (Piperidine) *Piper nigrum, Piper longum* 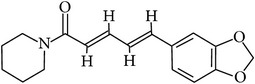	Swiss mice Acetic acid	↓ ulcer area and macroscopic score ↓ colon weight/ length ratio ↓ microscopic score and cell infiltration ↓ MPO activity and MDA content ↑ SOD activity and GSH levels ↓ TNF-*α* and NO levels ↓ free fat acids levels ↓ histological damage	[86]

** ** *Sanguinarine* (Isoquinoline) *Sanguinaria canadensis, Argemone mexicana* 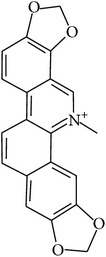	Kunming mice Acetic acid	↓ mortality rate and body weight loss ↓ DAI ↓ macroscopic damage score ↓ histological damage score ↓ IL-6 and TNF-*α* expression ↓ IL-6, TNF-*α* serum, and colonic levels ↓ MPO tissue accumulation	[87]

** ** *Sinomenine* (Isoquinoline) *Sinomenium acutum* 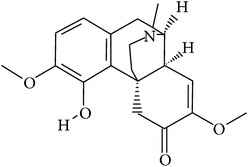	BALB/c mice TNBS	↓ body weight loss ↓ macroscopic and histological damage scores ↓ MPO colonic activity ↓ TNF-*α* and INF-*γ* mRNA and protein expression	[88]
	BALB/c mice TNBS	↓ body weight loss, mortality, and diarrhea ↓ histological damage score ↓ miR-155 in colon tissue ↓ c-Maf, TNF-*α*, and INF-*γ* mRNA expression and protein levels in colon	[89]

** ** *Skimmianine* (Quinoline) Pericarpium Zanthoxyli 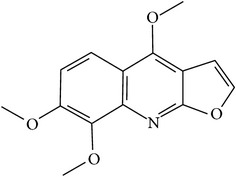	Sprague-Dawley rats TNBS	= colon index = ulcerative area ↓ LBP level in colon tissue = TNF-*α* levels in colon tissue ↓ NF-*κ*B expression in colon tissue ↓ TLR4 expression in colon tissue ↓ LBP level in LPS treated cells (HT29 cells) ↓ PGE_2_ level in LPS treated cells (HT29 cells) = TNF-*α* level in LPS treated cells (HT29 cells)	[56]

** ** *Sophocarpine* (Quinolizidine) *Sophora alopecuroides* 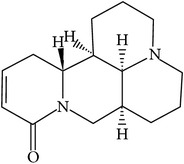	C57BL/6 mice DSS	↓ DAI ↓ weight loss ↓ colon length shortening ↓ histological damage score ↓ MPO colonic activity ↓ IL-6 and IL-1*β* serum levels	[90]

* Sophoridine* (Quinolizidine) *Sophora alopecuroides* 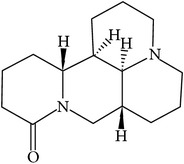	C57BL/6 mice DSS	↓ damage scores ↓ plasma haptoglobin ↓ ICAM-1 levels	[91]^*∗*^

** ** *Tetrandrine* (Isoquinoline) *Stephania tetrandra* 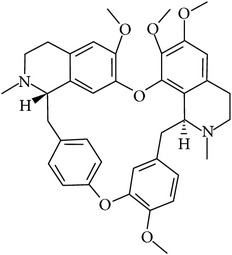	BALB/c mice DSS	↓ DAI and histological damage score ↓ TNF-*α* and IL-1*β* mRNA and protein expression ↓ NF-*κ*B DNA binding activity ↓ MPO colonic activity	[92]

** ** *Tryptanthin* (Indoloquinazoline) *Candida lipolytica, Couroupita guianensis, Isatis tinctoria, Wrightia tinctoria, Strobilanthes cusia * 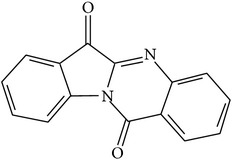	CBA mice DSS	↑ animal survival ↓ colon inflammation (MRI) ↓ thickness of bowel wall (MRI) ↓ intestinal microvasculature damage (MRI)	[93]

* W-8 (Tylophorine analog)* (Phenanthroindolizidine) Derivative of tylophorine, encountered in *Tylophora *sp. 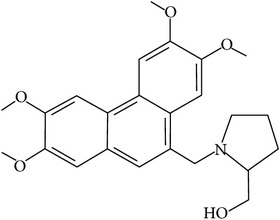	BALB/c mice TNBS	↑ Body weight ↑ Colon length ↓ histological damage score ↑ IL-10 and Fox3 mRNA colonic levels ↓ INF-*γ* and TNF-*α* mRNA colonic levels	[94]

↓ minor, ↑ major, = similar, when compared with control colitis.^*∗*^Only abstract accessed.^*∗∗*^Zhang et al. [61] also evaluted the alkaloids 13-Methyldihydrocoptisine, 8-(1-Acetylethenyl)-13-methylcoptisine Chloride, 8-(1-Propionylethenyl)-13-methylcoptisine Chloride, 13-(2,4-Difluorobenzyl)coptisine Chloride, 13-(2,4-Difluorobenzyl)-Dihydrocoptisine, Tetrahydrocoptisine, 8-oxodihydrocoptisine in DSS-induced colitis. They exhibited very weak efficacy or no efficacy in vivo (data not show in original paper).
